# Ecological value of macrophyte cover in creating habitat for microalgae (diatoms) and zooplankton (rotifers and crustaceans) in small field and forest water bodies

**DOI:** 10.1371/journal.pone.0177317

**Published:** 2017-05-04

**Authors:** Sofia Celewicz-Gołdyn, Natalia Kuczyńska-Kippen

**Affiliations:** 1 Department of Botany, Faculty of Horticulture and Landscape Architecture, Poznań University of Life Sciences, Poznań, Poland; 2 Department of Water Protection, Faculty of Biology, Adam Mickiewicz University, Poznań, Poland; Charles University, CZECH REPUBLIC

## Abstract

Due to their small area and shallow depth ponds are usually treated as a single sampling unit, while various microhabitats offer different environmental conditions. Thus, we tested the effect of different habitat types typically found within small ponds on the microalgae and zooplankton communities. We found that submerged macrophytes have the strongest impact on microalgae and zooplankton communities out of all the analysed habitats. Some epontic diatoms (e.g. *Fragilaria dilatata*, *Cymbella affinis*) and littoral-associated zooplankton species (e.g. *Simocephalus vetulus*, *Lecane bulla*) were significantly related to elodeids. However, pelagic species (e.g. bosminids) preferred less complex helophytes, which suggests that the most heterogeneous elodeid habitats were not an anti-predator shelter for cladocerans. Selection of different macrophyte types by taxonomically various organisms suggests that it is not only macrophyte cover that is desired for healthy aquatic environment but that a level of habitat mosaic is required to ensure the well-being of aquatic food webs. Species-specific preferences for different types of macrophytes indicate the high ecological value of macrophyte cover in ponds and a potential direction for the management of small water bodies towards maintaining a great variation of aquatic plants. Moreover, the type of surrounding landscape, reflecting human-induced disturbance (28 field ponds) and natural catchment (26 forest ponds), significantly influenced only zooplankton, while diatoms were affected indirectly through the level of conductivity. Nutrient overload (higher content of TRP) and increased conductivity in the field landscape contributed to a rise in microalgae (e.g. *Amphora pediculus*, *Gomphonema parvulum*) and zooplankton (e.g. *Thermocyclops oithonoides*, *Eubosmina coregoni*) abundance. An awareness of the responses of both components of plankton communities to environmental factors is necessary for maintaining the good state of small water bodies in various types of landscape.

## Introduction

Small water bodies have many important functions because they provide several ecosystem services, and increase not only local, but also regional biodiversity [1] due to their diverse flora and fauna, including rare, endemic and species of high conservation interest [2–5]. In spite of their small areal extent, small ponds play an important role in global cycles, particularly of nitrogen and phosphorus [6–7]. As the stability of a small water body ecosystem is often threatened by both warming climate conditions and human-induced pressure their ecological quality around the world is declining [8–10], which in turn leads to the disappearance of their inhabitants [2]. The high value of biological diversity associated with ponds suggests that they should be the principal target of strategies for the protection of aquatic biodiversity in Europe [11].

Ponds are subject to great fluctuations in abiotic characteristics due to their shallowness and small area [12], thus providing specific conditions for the inhabiting organisms. The critical elements in a pond’s food chain are microalgae and zooplankton. They quickly react to changes in physical and chemical parameters of water. Although microalgae (particularly diatoms) and zooplankton (particularly rotifers and cladocerans) are widely used as indicators in freshwater ecosystems [12–14], little is known about their communities in small water bodies. Ponds also provide varied microhabitats, particularly structured by aquatic vegetation of a mosaic pattern. Different factors are expected to determine microalgae and zooplankton communities among macrophytes when compared to the open water areas. It is well known that plant habitat complexity, understood as the morphological build of a plant, structures lake plankton [15–16]. Plants with dissected leaves (elodeids) provide organisms with more substrate for foraging and with more effective shelter from predators in comparison with undissected helophytes [17–18], which are less complex. Not much is known about the effect that aquatic vegetation has on habitat differentiation and on plankton occurrence in ponds. The available data on heleoplankton usually comes from temporal studies, in annual or long-term cycles [19–20], mainly concerning the open water area. Macrophytes also influence abiotic parameters [21] and have an impact on relationships between organisms in the aquatic food web [22]. The physical and chemical parameters of water are also influenced by the surrounding environment. The character of land use in the catchment area markedly transforms the water chemistry and is known to affect both the taxonomic structure and the dynamics of aquatic communities in lakes [23–25] or streams [26]. It can also be expected that the type of direct surroundings of small water bodies will alter the abiotic features of water and sediments and in turn microalgae and zooplankton.

The aim of the present study was to examine the effect of different habitat types found within small ponds on the microalgae and zooplankton communities. Considering all of the above-mentioned ecological aspects, we expect that the habitat heterogeneity within a pond to be of great importance, despite the fact that small water bodies are simple ecosystems with limited morphological features. We will also analyse and discuss the role of the catchment area (forest vs. field) and associated physical and chemical factors of the water on the microalgae and zooplankton assemblages.

## Methods

### Locality description and sampling sites

Investigations were conducted in 54 small water bodies, located in the Wielkopolska Lakeland (Western Poland) (see the S1 File), during the optimum phase of the vegetation season (June and July).

All analyses included small water bodies situated in two types of catchment area. In total 28 ponds were qualified as typically field and 26 as forest ponds. Due to the fact that the direct character of land use and the specificity of the buffer strip have a very strong effect on the water quality and consequently on the functioning of the inhabiting organisms [27], we analysed the type of surroundings of each pond. The potential pressure of the catchment on a group of pastoral ponds in the Wielkopolska region, where our examination was conducted, was high, amounting to a mean Ohle index >140 [28]. All forest ponds were located in 100% forest catchment. Field ponds were regularly under strong anthropogenic impact, with surroundings dominated by typically arable land.

Sampling stations were located within three types of habitats: elodeids (35 stations), helophytes (23 stations) and the open water (52 stations: 26 in field and 26 in forest ponds) of the investigated ponds.

In two groups of ponds (field and forest) all available microhabitats (the open water area and macrophyte sites) were analysed. However, due to various environmental factors, e.g. generally higher degree of shading, fewer macrophyte-dominated habitats were analysed in forest ponds (14 elodeid stations and 9 helophyte stations) compared to field ponds (21 and 14, respectively).

### Physical and chemical analyses

Dissolved oxygen, pH and conductivity (reflecting the total amount of dissolved ions in the water) were measured directly using a Portable Multiparameter Meter Sension 156 Hach (Hach Co., USA) at the sampling sites. Chemical analyses were conducted in the laboratory following standard methods [29] in order to determine total reactive phosphorus (TRP), nitrate (NO_3_), nitrite (NO_2_), ammonium (NH_4_) and total hardness (CaCO_3_). Dissolved inorganic nitrogen (DIN) concentration was calculated by summing the concentration of nitrate, nitrite and ammonium. The chlorophyll *a* content was determined with a spectrophotometer (at 663 and 750 nm), following extraction in 4°C acetone [30].

### Macrophytes

Aquatic plants of the examined water bodies represented two ecological groups: submerged macrophytes—elodeids (*Ceratophyllum demersum* L., *Ceratophyllum submersum* L., *Chara hispida* L., *Chara tomentosa* L., *Myriophyllum vericillatum* L., *Myriophyllum spicatum* L., *Nitellopsis obtusa* (Devs.) J. Groves, *Potamogeton lucens* L., *Potamogeton pectinatus* L.) and emerged macrophytes—helophytes (*Phragmites australis* (Cav.) Trin. ex Steud, *Typha angustifolia* L., *Typha latifolia* L., *Schoenoplectus lacustris* (L.) Palla). They formed separate habitats for planktonic organisms. To avoid overlapping of various habitats, the chosen beds of plants were fully representative monospecies phytocoenoses.

### Microalgae and zooplankton analyses

Microalgae and zooplankton were taken from each site in triplicate (total n = 330), using a plexiglass core sampler (∅ 50 mm; length 1.5 m) from among vegetated stations. In the open water area, the material was sampled using a calibrated vessel. Subsamples of *ca*. 1–2 L were taken from randomly selected places within each habitat to make up a 10 L sample. Microalgae samples were first fixed in Lugol solution and then preserved in formaldehyde. Samples for taxonomical and quantitative analyses were sedimented in the laboratory and thickened to a volume of 10 ml. Microalgae and zooplankton composition was determined with a light microscope (magnification 400x). Abundance of microalgae cells was counted over at least 160 fields of a Fuchs–Rosenthal chamber (Brand GmbH+CO KG, Wertheim, Germany). The zooplankton samples were concentrated using a 45 μm mesh net and fixed with 4% formalin. Rotifer and crustacean species were first determined and then counted in a 1.0 ml chamber, which was equal to 1 L of pond water. Representatives of Bdelloidea within rotifers were all counted, but not determined to a particular species.

### Statistical analyses

In order to determine whether there is a significant difference in the number of three types of habitats (elodeids, helophytes, the open water zone) between two types of pond—forest and field the λ^2^ test was applied. Differences in environmental factors and also in the mean abundance of microalgae and zooplankton between the two types of water bodies (Student’s t-test) and three types of habitats (ANOVA) were examined with Statistica v. 10 Software (StatSoft Inc., Tulsa, OK). Where significant effects were identified, post hoc analyses (the posteriori Tukey test) were undertaken.

In order to identify the relationship between particular environmental variables, including habitat (the open water zone, elodeids and helophytes), pond type (field/pastoral and forest), physical and chemical parameters of water within each analysed habitat (dissolved inorganic nitrogen–DIN, total reactive phosphorus–TRP, dissolved oxygen, conductivity, pH, water hardness) as well as microalgae and zooplankton abundance, Canonical Correspondence Analysis (CCA) was applied to the log transformed data with CANOCO 4.5 statistical computing environment software [31]. Data on species abundance were introduced to the models as dependent variables, while measured environmental factors were considered as explanatory variables. Forward selection of environmental variables was performed to find which of them add to the model significantly. The Monte Carlo Permutation Test (with 5000 replicates) was used on explanatory variables as well as on the canonical axes to evaluate the statistical significance of relationships between environmental and species data. CCA analyses were carried out using only taxa of highest frequency (microalgae species occurring in >19% and zooplankton species occurring in >25% of the whole set of samples) and/or dominating species (species that exceeded 10% of the total abundance of microalgae and zooplankton communities). The following taxonomical groups of microalgae were included, and also analysed separately in CCA analyses: cyanobacteria, chlorophytes, diatoms, euglenophytes, dinophytes and cryptophytes. They were tested using the Monte Carlo permutation test with the dependent variables containing numbers of individual species. To analyse the relationship between the abundance of diatoms and zooplankton species (included in the CCA analyses) and physical and chemical parameters of water, the Spearman correlation coefficients were calculated. For species that significantly differed in reference to environmental factors scatter plots (of the variables identified as significant), plotted against the abundance of zooplankton and diatom species, were performed. The significance of the relationships between particular plankton species abundance and the pond type were determined by the Mann-Whitney U-test. In order to examine the relationships between species data and habitat (elodeids, helophytes and the open water) the non-parametric Kruskal-Wallis test was used. These analyses were performed using Statistica 6.0 PL 2002 software (StatSoft Inc., Tulsa, OK). A p value of <0.05 was selected as the minimum level determining significance in all the statistical analyses.

The work did not involve any endangered or protected biological species. No specific permission was required for any of these locations and activities.

## Results

### Physical and chemical variables, microalgae and zooplankton in different types of habitats (open water, elodeids and helophytes)

The number of different habitat types was reasonably evenly distributed between two pond types (λ^2^ = 1.192, df = 2, p = 0.55).

The open water area of examined ponds was characterized by higher concentrations of nutrients (TRP and DIN) and chlorophyll a content compared to macrophyte-dominated stations (elodeids and helophytes). Moreover, rotifers prevailed in the open water zone. The remaining groups of plankton reached higher abundance in macrophyte-dominated areas. Crustaceans and diatoms had their highest densities among elodeids, while mean microalgae abundance was highest among helophytes (Table 1, S1 File).

**Table 1:** Limnological parameters and microalgae and zooplankton community abundance (Min-Max, Mean $\bar{x}$ ± SD) of three types of habitats (open water zone–water, elodeids, helophytes). The level of significance (p) of the analysis of variance (ANOVA) between the three types of habitats is given. The results of posteriori Tukey test in S1.

Type of habitat		Water			Elodeids			Helophytes			
Parameter	Unit	$\bar{x}$	Range	SD	$\bar{x}$	Range	SD	$\bar{x}$	Range	SD	p
pH		7.93	6.4–10.8	0.88	8.00	6.1–9.8	0.81	8.20	7.2–9.6	0.69	-
Conductivity	μS cm^-1^	731	26–1728	419	742	109–1736	392	759	116–1587	412	-
O_2_	%	88	5–259	53	92	3–224	51	89	28–175	36	-
TRP	μg P l^-1^	303	1–2181	527	233	3–1323	377	180	2–1213	333	-
DIN	mg l^-1^	2.2	0.7–9.1	1.7	1.5	0.5–3.1	0.5	1.3	0.6–3.2	0.6	**
Hardness	mg l^-1^ CaCO_3_	321	9–1512	255	338	45–811	196	304	14–688	217	-
Chlorophyll *a*	μg l^-1^	72	0.1–2031	291	19	1–240	41	18	1–81	22	-
Diatoms	mln ind l^-1^	0.3	0–3	0.6	0.7	0–7.1	1	0.5	0.0002–6	1	-
Microalgae	mln ind l^-1^	7.2	3–157	22	3	0.01–13	3	12	0.09–100	26	-
Rotifera	ind l^-1^	3864	5–42655	8803	2492	10–27889	4879	3499	3–19095	5329	-
Crustacea	ind l^-1^	147	1–1991	347	709	3–3960	1050	540	9–4128	1113	**

*—p<0.05

**—p<0.01

***—p<0.001.

### Physical and chemical variables, microalgae and zooplankton in two types of ponds (forest vs. field)

The level of conductivity, TRP and hardness were significantly higher in field ponds, while oxygen saturation was lower in this type of water body. Field ponds were characterised by a higher mean abundance of both groups of zooplankton and microalgae compared with forest water bodies, while diatoms were more abundant in forest ponds (Table 2).

**Table 2:** Limnological parameters and microalgae and zooplankton community abundance (Min-Max, Mean ± SD) of two types of ponds (TRP–total reactive phosphorus; DIN–dissolved inorganic nitrogen). The level of significance (p) of the t-test between the two types of water bodies is given.

Type of pond		Forest water bodies			Field water bodies			
Parameter	Unit	$\bar{x}$	Range	SD	$\bar{x}$	Range	SD	p
pH		7.88	6.3–10.0	0.87	8.14	6.6–10.8	0.82	-
Conductivity	μS cm^-1^	483	26–1085	295	899	109–1736	407	***
O_2_	%	103	22–259	54	79	3–178	44	**
TRP	μg P l^-1^	78	1–590	126	379	3–2128	536	***
DIN	mg l^-1^	1.7	0.7–6.2	1.0	1.9	0.5–9.1	1.5	-
Hardness	mg l^-1^ CaCO_3_	209	9–530	134	407	45–1512	247	***
Chlorophyll *a*	μg l^-1^	30	0.1–259	59	64	0.5–2031	266	-
Diatoms	mln ind l^-1^	0.7	0–7	1	0.3	0–3	0.5	*
Microalgae	mln ind l^-1^	4	3–29	6	9	0.01–157	25	-
Rotifera	ind l^-1^	2409	3–13356	3105	4108	5–42655	9023	-
Crustacea	ind l^-1^	244	1–2720	477	555	1–4128	1051	*

*—p<0.05

**—p<0.01

***—p<0.001.

### Diatoms vs. environmental variables

**Fig 1:**
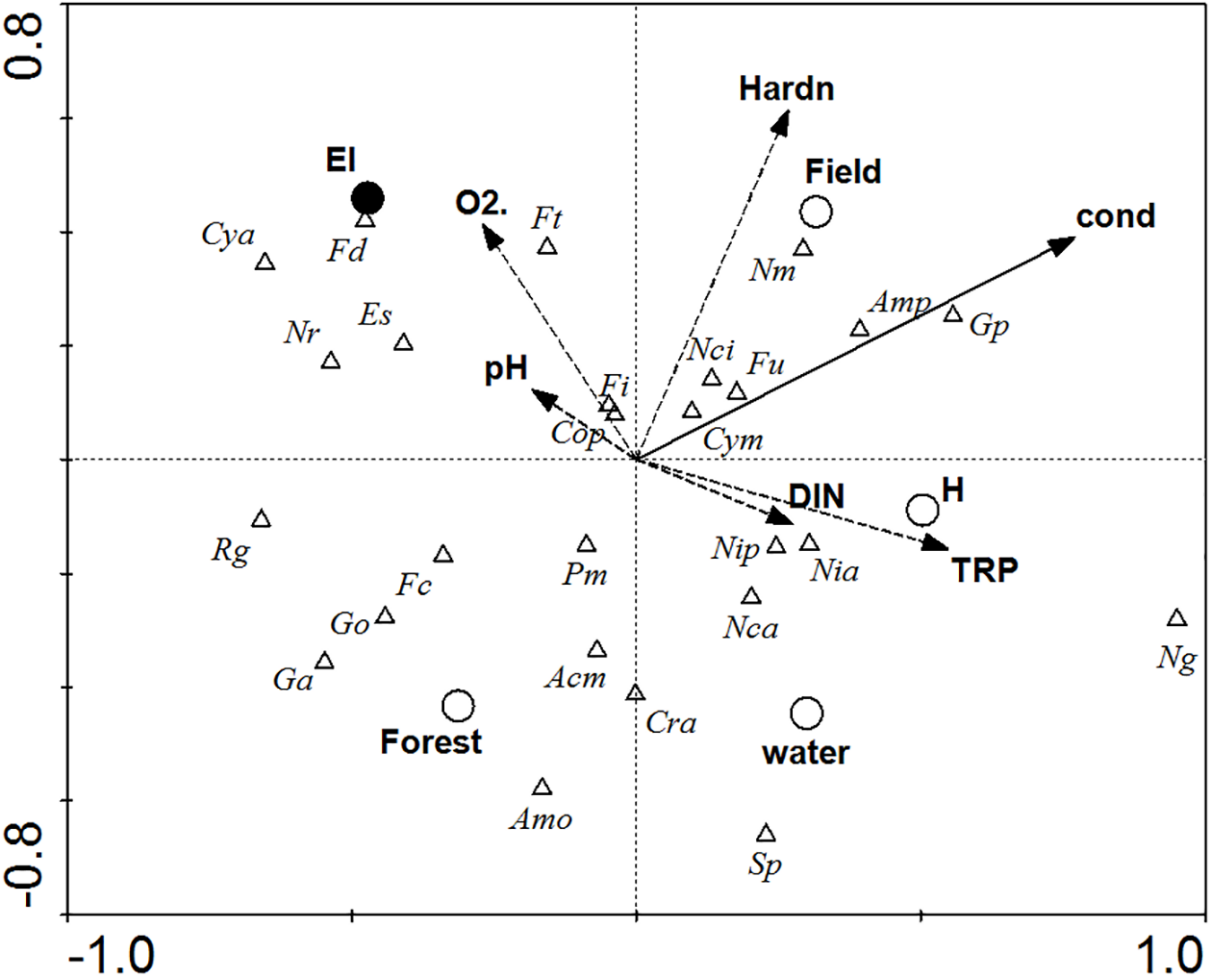
Canonical Correspondence Analysis (CCA) diagram showing relations between the abundance of diatom species (triangles) and environmental factors studied (arrows: linear variables; circles: binominal variables). Solid lines and filled circles: variables significantly adding to the model according to Forward selection with Monte Carlo permutation test (p < 0.05); dashed lines and open circles: non-significant variables. The whole model is significant at p < 0.001, F = 1.463; eigenvalues: horizontal (I) axis = 0.136; vertical (II) axis = 0.078. **Diatom species**: Acm–*Achnanthes minutissima* var. *affinis* (Grun.) Lange-Bertalot, Amo–*Amphora ovalis* (Kütz.) Kütz., Amp–*Amphora pediculus* (Kütz.) Grunow, Cop–*Cocconeis placentula* Ehrenb., Cra–*Cyclotella radiosa* (Grun.) Lemm., Cya–*Cymbella affinis* Kütz, Cym–*Cymbella minuta* Hilse ex Rabenhorst, Es–*Epithemia sorex* Kütz., Fc–*Fragilaria capucina* Desm., Fd–*Fragilaria dilatata* (Bréb.) Lange-Bertalot, Fi–*Fragilaria intermedia* Grun., Ft–*Fragilaria tenera* (Smith) Lange-Bertalot, Fu–*Fragilaria ulna* (Nitzsch) Lange-Bertalot var. *ulna*, Ga–*Gomphonema acuminatum* Ehrenberg, Go–*Gomphonema olivaceum* Kütz., Gp–*Gomphonema parvulum* (Kütz.) Kütz., Nca–*Navicula capitata* var. *hungarica* (Grunow) Ross, Nci–*Navicula cincta* (Ehrenberg) Ralfs, Ng–*Navicula gracilis* Ehrenberg, Nm–*Navicula menisculus* Schumann, Nr–*Navicula radiosa* Kütz., Nia–*Nitzschia acicularis* (Kütz.) W. Smith, Nip–*Nitzschia palea* (Kutz.) W. Smith, Pm–*Pinnularia maior* (Kütz.) Cl., Rg–*Rhopalodia gibba* (Ehr.) O. Müll., Sp–*Stauroneis phoenicentron* Ehr.

From all the taxonomic groups analysed with CCA, only diatom communities were significantly affected by environmental variables, based on the distribution of the 26 most frequently encountered taxa (Fig 1, Table 3).

**Table 3:** Results of Canonical Correspondence Analysis (CCA) on relations between the abundance of diatom species and environmental factors studied. Values of P and F are calculated using Monte Carlo permutation test with 5000 permutations.

Variable	Abbreviations on CCA diagram	Variance explained (%)	P	F
**Water conductivity**	**cond**	**10**	**< 0.001**	**3.12**
**Elodeids**	**El**	**5**	**0.030**	**1.64**
Field or Forest catchment	Field / Forest	5	0.053	1.55
Dissolved oxygen contents	O_2_	5	0.061	1.55
Water hardness	Hardn	5	0.068	1.54
Dissolved inorganic nitrogen	DIN	3	0.237	1.20
Water reactivity	pH	3	0.554	0.92
Open water / Helophytes	Water / H	3	0.709	0.80
Total reactive phosphorus	TRP	2	0.725	0.78
Whole model		41	< 0.001	1.463

Bold = variables significantly adding to the model at p < 0.05 level.

The model explained 41% of the variance and was significant at the level p<0.001. The results of CCA (Table 3) showed that conductivity was the most significant environmental determinant influencing diatom community structure in the examined ponds. The species associated with higher values of conductivity were *Gomphonema parvulum*, *Amphora pediculus*, *Navicula menisculus*, *Fragilaria ulna*, *Navicula cincta* and *Cymbella minuta*. Species negatively related to conductivity were *Gomphonema acuminatum*, *Gomphonema olivaceum*, *Fragilaria capucina*, *Pinnularia maior* and *Rhopalodia gibba*. According to the model (Fig 1), the type of habitat (elodeids) was another factor explaining the structure of diatom assemblages. Species such as *Cymbella affinis*, *Fragilaria dilatata*, *Navicula radiosa*, *Epithemia sorex* and *Fragilaria tenera* were associated with elodeids, while *Navicula gracilis* was distinctly negatively related to this type of habitat. The other variables included (Table 3) had no significant effect (p>0.05).

### Zooplankton vs. environmental variables

**Fig 2:**
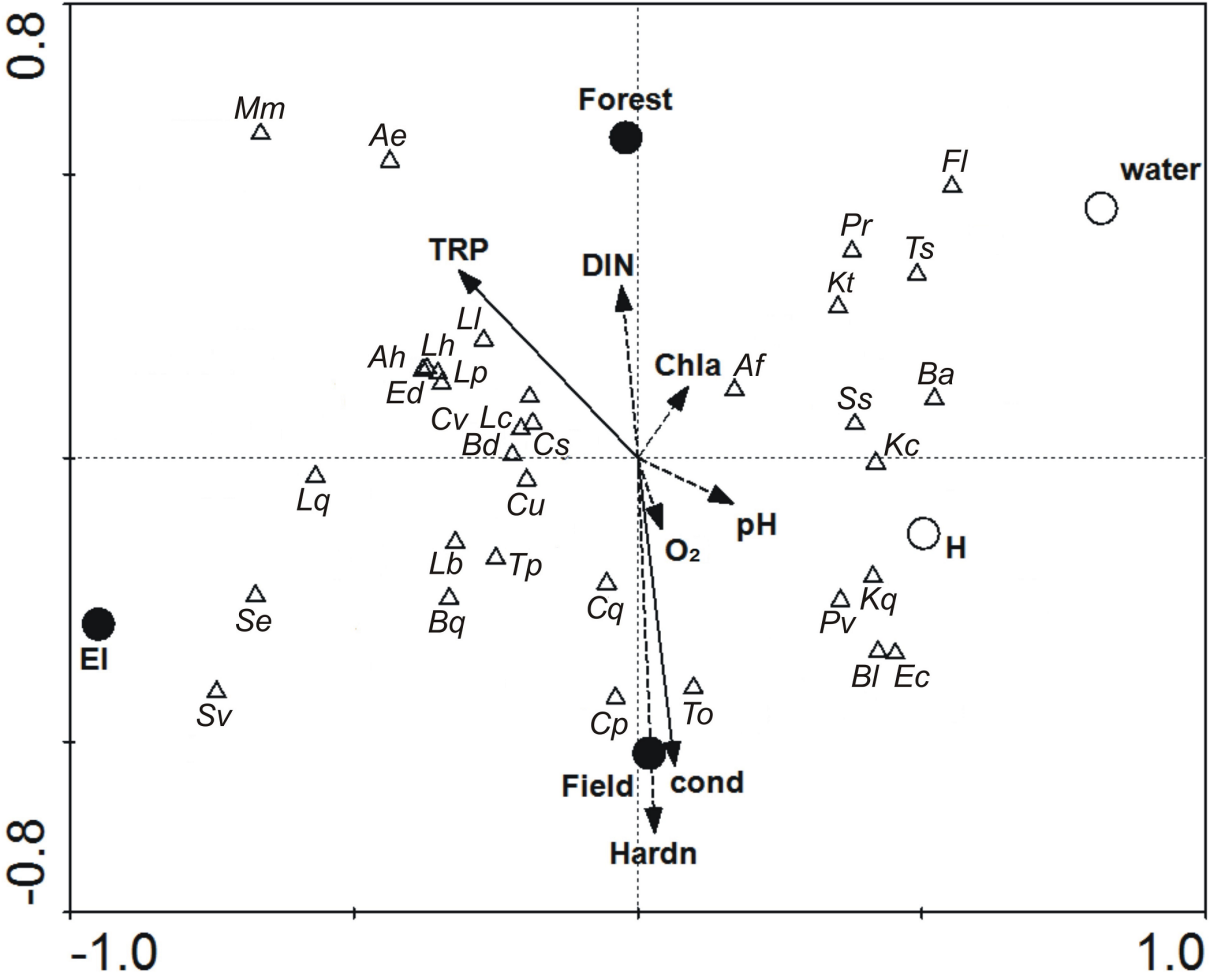
Canonical Correspondence Analysis (CCA) diagram showing relations between the abundance of zooplankton species (triangles) and environmental factors studied (arrows: linear variables; circles: binominal variables). Solid lines and filled circles: variables significantly adding to the model according to Forward selection with Monte Carlo permutation test (p < 0.05); dashed lines and open circles: non-significant variables. The whole model is significant at p < 0.001, F = 3.071; eigenvalues: horizontal (I) axis = 0.153; vertical (II) axis = 0.062. **Rotifera species**: Af–*Anuraeopsis fissa* (Gosse), Bd–*Bdelloidae*, Ba–*Brachionus angularis* Gosse, Bq–*Brachionus quadridentatus* (Hermann), Cv–*Cephalodella ventripes* Dixon-Nuttall, Cu–*Colurella uncinata* (O.F. Müller), Ed–*Euchlanis dilatata* Ehrenberg, Fl–*Filinia longiseta* (Ehrenberg), Kc–*Keratella cochlearis* (Gosse), Kt–*Keratella cochlearis* f. *tecta* (Lauterborn), Kq–*Keratella quadrata* (O.F. Müller), Lb–*Lecane bulla* (Gosse), Lc–*L*. *closterocerca* (Schmarda), Lh–*L*. *hamata* (Stoces), Ll–*Lecane lunaris* (Ehrenberg), Lp–*Lepadella patella* (O.F. Müller), Lq–*Lepadella quadricarinata* (Stenroos), Mm–*Mytilina mucronata* (O.F. Müller), Pr–*Polyarthra remata* (Skorikov), Pv–*Polyarthra vulgaris* Carlin, Ss–*Synchaeta* sp., Tp–*Testudinella patina* (Hermann), Ts–*Trichocerca similis* (Wierzejski). **Crustacea species**: Ah–*Acroperus harpae* (Baird), Ae–*Alonella excisa* (Fischer), Ec–*Euosmina coregoni* Baird, Bl–*Bosmina longirostris* (O.F. Müller), Cq–*Ceriodaphnia quadrangula* (O.F. Müller), Cp–*Ceriodaphnia pulchella* Sars, Cs–*Chydorus sphaericus* (O.F. Müller), Se–*Simocephalus exspinosus* (Koch), Sv–*Simocephalus vetulus* (O.F. Müller), To–*Thermocyclops oithonoides* (Sars).

Analysing relations between zooplankton and environmental factors, the explanatory variables describing type of habitat were located along the first axis, whereas the second axis mostly described physical and chemical parameters associated with the type of catchment area (Fig 2).

The model explained 35.6% of the variance and was significant at the level p<0.001. The CCA diagram indicated that the key factor that influenced zooplankton communities was the presence of elodeids (Fig 2, Table 4).

**Table 4:** Results of Canonical Correspondence Analysis (CCA) on relations between the abundance of zooplankton species and environmental factors studied. Values of P and F are calculated using Monte Carlo permutation test with 5000 permutations.

Variable	Abbreviations on CCA diagram	Variance explained (%)	P	F
**Elodeids**	**El**	**13**	**< 0.001**	**9.48**
**Total reactive phosphorus**	**TRP**	**5**	**< 0.001**	**4.08**
**Water conductivity**	**cond**	**5**	**< 0.001**	**4.01**
**Field or Forest catchment**	**Field / Forest**	**4**	**0.003**	**2.54**
Dissolved inorganic nitrogen	DIN	2	0.068	1.61
Water hardness	Hardn	2	0.063	1.64
Open water / Helophytes	Water / H	2	0.068	1.59
Water reactivity	pH	1	0.202	1.27
Dissolved oxygen contents	O_2_	2	0.282	1.16
Whole model		36	< 0.001	3.071

Bold = variables significantly adding to the model at p < 0.05 level.

The species associated with this type of habitat were *Simocephalus* species (*S*. *exspinosus* and *S*. *vetulus*), *Brachionus quadridentatus*, *Lecane bulla*, *Testudinella patina* and to a lesser extent *Ceriodaphnia* species (*C*. *pulchella* and *C*. *quadrangula*). The second group of species gathered around the open water area and was negatively related to elodeids (e.g. *Filinia longiseta*, *Polyarthra remata*, *Trichocerca similis*, *Keratella cochlearis* f. *tecta*, *Brachionus angularis* or *Anuraeopsis fissa*). Other significant factors that had an impact on zooplankton communities were TRP and water conductivity (Table 4). According to the results, TRP positively influenced the abundance of species such as *Mytilina mucronata*, *Alonella excisa*, *Lecane lunaris*, *Lecane hamata*, *Lepadella patella*, *Acroperus harpae* or *Euchlanis dilatata*, while some other species (*Thermocyclops oithonoides*, *Eubosmina coregoni*, *Bosmina longirostris*, *Polyarthra vulgaris* and *Keratella quadrata*) were related to conductivity. At the same time, these species were attributed to helophytes. The type of catchment area was also among the significant features affecting zooplankton species and explained 4% of the variance of the CCA diagram. Field catchment around ponds was associated with the group of species affected by an increasing level of conductivity, while species associated with TRP were recorded in forest ponds (Fig 2). Other variables included in the canonical analysis did not improve the model significantly (p>0.05).

Non-parametric statistics (data available in S1 File) supported the significance of the relations between the diatom and zooplankton species and the environmental variables, pond type and habitat type.

## Discussion

### The role of habitat type in structuring diatom and zooplankton assemblages

As we expected, the type of habitat was a significant factor structuring both microalgae and zooplankton communities within the 54 analysed small water bodies, similarly to larger aquatic ecosystems. The results of statistical analyses showed that the variation of both microalgae and zooplankton species was distinctly affected by the presence of submerged macrophytes (elodeids), one of the three studied types of habitat, irrespective of the type of water body (field vs. forest).

The only group of microalgae significantly affected by the environmental variables was diatoms. It is well known that diatoms, owing to their sensitivity to environmental changes, indicate a certain level of water quality in many aquatic systems [32–35, 12]. Diatom taxa associated with elodeids were sessile (epontic and/or benthic) forms, often typical of shallow water bodies [36–38] with high water mixing, e.g. *Cymbella affinis*, *Fragilaria dilatata*, *Epithemia sorex*, *Navicula radiosa* [39]. Elodeids create a habitat of the highest level of heterogeneity characterised by the greatest spatial and morphological complexity, measured by the density of plant stems [40]. Thus, elodeids provided a favourable environment for different diatom life forms: epontic (live attached to the substrate), and epontic/benthic or tychoplanktonic (occur in plankton, but are of epontic origin), e.g. *Fragilaria capucina* [39]. Results presented by Simkhada & Jüttner [41] also confirm a higher abundance of diatoms related to zones with submerged vegetation, similarly to our findings. Several authors have suggested that the elodeids and algae could compete for light and/or nutrients or that allelopathic mechanisms between both groups of primary producers may also exist [42–45]. This may explain why some diatoms, e.g. *Navicula gracilis*, were negatively correlated to submerged plants.

Some zooplankton taxa also preferred elodeids, such as cladocerans *Simocephalus exspinosus*, *S*. *vetulus*, *Ceriodaphnia pulchella* or *C*. *quandrangula*; and rotifers *Brachionus quadridentatus*, *Lecane bulla* and *Testudinella patina*. Most of the crustaceans were associated with macrophyte habitats, while in the case of rotifers, only the littoral species were present there. Elodeids supported a variable community of littoral zooplankters, whereas pelagic crustaceans (e.g. bosminids) preferred less complex helophytes, possibly treating them as an anti-predator refuge. This suggests that crustaceans find favourable life conditions among aquatic plants (littoral forms) as well as concealment from predators (pelagic forms) [45]. Moreover, spatial segregation may also express the different feeding requirements and/or swimming behaviour of littoral and pelagic cladocerans [46]. Therefore, a mosaic of different habitats within the ponds is necessary to support the co-existence of organisms with different habitat preferences. A third group of zooplankton (incl. *Anuraeopsis fissa*, *Brachionus angularis*, *Filinia longiseta*, *Keratella cochlearis* f. *tecta*, *Polyarthra remata* and *Trichocerca similis*) also emerged in our analysis. This group, with first four species being indicators of eutrophy [47, 48], preferred the open water area with prevailing high values of TRP, DIN and chlorophyll a. These species were found in opposition to elodeids, which confirms that eutrophication may be responsible for the disappearance of submerged macrophytes and the switch to a turbid and phytoplankton-dominated state [49], dominated by small-bodied zooplankton [50]. On the other hand, submerged macrophytes intensively uptake nutrients from the water column, and in this way purify water quality in shallow water bodies [51–52]. Thus zooplankton species that prefer eutrophy are not found in relation to elodeids.

### The impact of the catchment area and associated physical and chemical factors on microalgae and zooplankton communities

We have demonstrated that the water quality associated with the type of surrounding landscape significantly influenced the structure of microalgae and zooplankton assemblages. The high abundance of microalgae and zooplankton observed in field ponds was associated with the higher values of nutrients (DIN and TRP) and conductivity in the more fertilized water bodies. Meanwhile, the abundance of diatoms was significantly higher in forest ponds, which could be a result of the higher overshading here. It is well known that periphytic diatom species associated with macrophytes (such as dominating species in our ponds) are low light tolerant. They are well-adapted to low light levels and resistant to shading caused by plant cover [53]. Shade-tolerant diatoms could therefore, in the forest ponds win the competition for a niche with representatives of other groups of microalgae which did not have such a high tolerance to light shortage.

Among the physical and chemical parameters, only water conductivity significantly influenced the structure of both diatoms and zooplankton. Conductivity significantly lower in forest ponds, particularly increased the abundance of diatoms such as *Gomphonema acuminatum* and *Gomphonema olivaceum*. On the other hand, higher conductivity in field ponds was responsible for the increase of individual numbers of *Amphora pediculus* and *Gomphonema parvulum* and of some zooplankton species (*Thermocyclops oithonoides*, *Eubosmina coregoni*, *Bosmina longirostris*, *Polyarthra vulgaris* and *Keratella quadrata*). Therefore, our results show that agricultural practices in the surroundings of a pond increase the level of conductivity, which is in accordance with other studies carried out on wetlands [54, 9]. Furthermore, Rydén et al. [55] stated that the increasing proportion of cultivated land, with a higher level of fertilisation of usually fine-grained soil, leads to a large transport of all kind of ions. Some literature data concerning small water bodies [21] have also demonstrated that electric conductivity decreases in the presence of submerged vegetation, although we did not obtain any significant variation in conductivity level between microhabitats. Evidence of overfertilisation was additionally enhanced by a notably higher content of TRP in field ponds, which significantly structured zooplankton in our study.

## Conclusions

The type of habitat, together with water quality connected with the type of catchment, were of high significance. Elodeids had a strong influence on the community structure of both diatoms and zooplankton in a direct way (1. creating favourable conditions and substratum for sessile species; and 2. inhibiting the occurrence of some species through the release of allelopathic compounds). The diverse type of habitat preferences of cladoceran species–elodeids with littoral-associated cladocerans and helophytes with pelagic species (e.g. bosminids)–may suggest that helophytes serve as a refuge against predators. But such spatial differentiation may also indicate different feeding modes and/or swimming behaviour of littoral and pelagic taxa. This fact highlights the need to maintain within the area of a small water body a high complexity of macrophyte cover so as to allow the co-existence of organisms with different habitat requirements. In addition, the type of catchment area had an impact on diatoms and zooplankton in an indirect way, by conditioning the physical and chemical parameters of water.

The novelty of our study is that it has been shown that in small water bodies, similarly to large aquatic systems such as lakes, co-occurrence of various habitats substantially determine the structure of both diatoms and zooplankton, despite the small depth and surface area of ponds. What is more, the mosaic of habitats not only increases overall biodiversity but should also be a key element in conservation management.

## Supporting information

S1 File Geographical coordinates, limnological parameters and abundance of the most frequent and/or dominant zooplankton and diatom taxa in the sampling sites. The relationships between the abundance of diatom and zooplankton species and environmental parameters, types of water bodies and habitat types.
(XLSX)
